# Supplementary material for: Association between oral microbiome and seven types of cancers in East Asian population: a two-sample Mendelian randomization analysis
Source: Front Mol Biosci. 2023 Nov 21;10:1327893. doi: 10.3389/fmolb.2023.1327893 (PMC10702768; doi:10.3389/fmolb.2023.1327893)
Supplement: Supplementary file 1 [file DataSheet1.ZIP › Supplementary Materials/MR plots of saliva/saliva═╝/ovarian cancer/pheno.2873_to_ovarian cancer_leave_one_out.pdf]

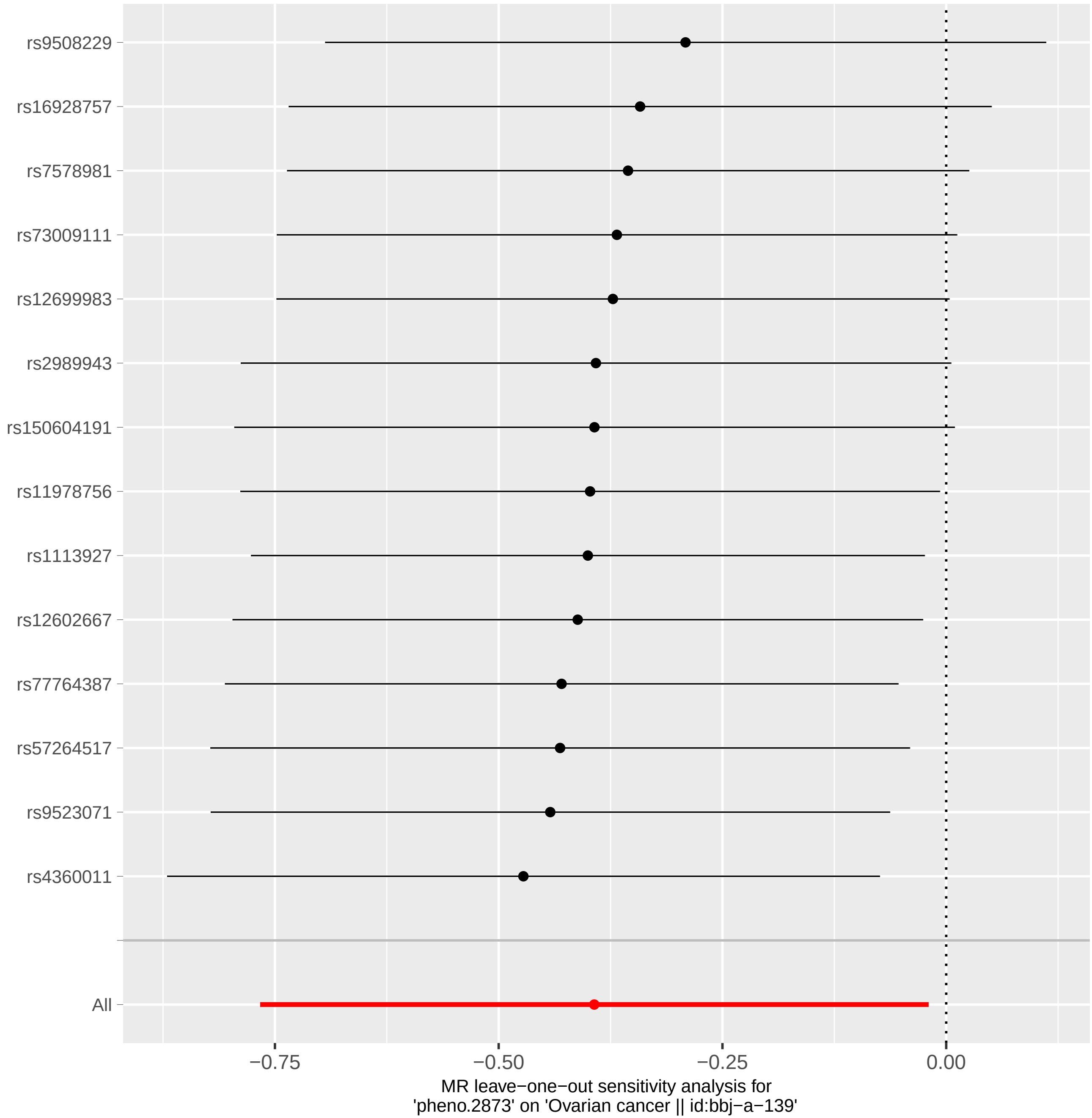

rs9508229
rs16928757
rs7578981
rs73009111
rs12699983
rs2989943
rs150604191
rs11978756
rs1113927
rs12602667
rs77764387
rs57264517
rs9523071
rs4360011
All
−0.75
−0.50
−0.25
0.00
MR leave−one−out sensitivity analysis for
'pheno.2873' on 'Ovarian cancer || id:bbj−a−139'
